# Duodenal Stenosis: A Diagnostic Challenge in a Neonate With Poor Weight Gain

**DOI:** 10.7759/cureus.8559

**Published:** 2020-06-11

**Authors:** Ma Khin Khin Win, Carole Mensah, Kunal Kaushik, Louisdon Pierre, Adebayo Adeyinka

**Affiliations:** 1 Pediatrics, The Brooklyn Hospital Center, Brooklyn, USA

**Keywords:** duodenal stenosis, neonate, pediatric, duodenostomy, duodenal atresia, hypochloremia, metabolic alkalosis

## Abstract

Cases of isolated duodenal stenosis in the neonatal period are minimally reported in pediatric literature. Causes of small bowel obstruction such as duodenal atresia or malrotation with midgut volvulus have been well documented and are often diagnosed due to their acute clinical presentation. Duodenal stenosis, however, causes an incomplete intestinal obstruction with a more indolent and varying clinical presentation thus making it a diagnostic challenge. We present a neonate with a unique case of congenital duodenal stenosis. The neonate presented with poor weight gain and frequent "spit-ups" as per the mother at the initial newborn visit. The clinical presentation was masked as the patient was being fed infrequently and with concentrated formula. We postulate that this may be due to the fact that the mother was an adolescent and relatively inexperienced with newborn care. During the hospital course, the patient had recurrent episodes of emesis with notable electrolyte abnormalities including hypochloremia and metabolic alkalosis. Further investigation with an abdominal X-ray showed dilated loops of bowel. Pyloric stenosis was ruled out via abdominal ultrasound. An upper gastrointestinal (GI) series ultimately confirmed a diagnosis of duodenal stenosis and the infant underwent surgical repair with full recovery. Congenital duodenal stenosis may have atypical presentations in neonates requiring pediatricians to have a high index of suspicion for diagnosis and to ensure timely therapy.

## Introduction

The etiology of small bowel obstruction in the neonatal period is precipitated by a variety of pathologies, however, isolated duodenal stenosis in the newborn patient is a condition that has been marginally discussed in the pediatric literature. The literature on congenital duodenal obstruction has heavily focused on duodenal atresia and associated chromosomal anomalies [1]. In addition, reports of duodenal stenosis, which have not been precipitated by extrinsic factors such as annular pancreas or mesenteric vasculature compression, are even more sparse. Furthermore, duodenal stenosis is a cause of small bowel obstruction that often presents with symptoms more indolent in nature due to the partial blockage, making it a diagnostic challenge. While the typical picture of small bowel obstruction in the neonate has been documented in the literature, it is essential to remember that each patient is unique and may deviate from the classic presentation. Thus, we present an atypical case of small bowel obstruction in the neonate due to isolated congenital duodenal stenosis.

## Case presentation

A 13-day-old male child born full-term, weight appropriate for gestational age via normal spontaneous vaginal delivery was brought by the mother to our outpatient clinic for a follow-up visit. The neonate was found to have a 20% weight loss from his birth weight, which was quite significant. The infant was born to a teenage mother who received routine prenatal care. The mother’s laboratory results were negative except for gonorrhea and chlamydia, which were treated prior to delivery. The infant was exclusively formula-fed every 5-6 hours with a concentrated formula (2 scoops of formula powder mixed with 3 oz of water). The mother also reported "spit-ups" following feeds. The child had one to two bowel movements every other day and five to six wet diapers daily. Physical examination was significant for a cachectic appearing neonate with no subcutaneous fat appreciated. The abdomen was soft, non-distended, without palpable masses and bowel sounds were appreciated on auscultation. The child was subsequently admitted to the pediatric floor for further investigation and management due to his inadequate weight gain with clinical signs of malnutrition.

On the pediatric floor, the neonate was clinically stable with normal vital signs. Upon further investigation, the mother reported that the child had been vomiting “yellow” tinged partially digested milk, about one to two episodes daily since he started feeding. On the night of admission, the patient initially had one episode of non-bilious, non-bloody vomitus with partially digested formula after feeding. Initial laboratory testing which included a basic metabolic panel was significant for hypochloremic metabolic alkalosis (Table 1). 

**Table 1:** Initial basic metabolic panel

Lab	Value
Sodium (Na)	135 mmol/L (Normal: 136-145 mmol/L)
Chloride (Cl)	85 mmol/L (Normal: 98-107 mmol/L)
Potassium (K)	4.6 mmol/L (Normal: 3.5-5.1 mmol/L)
Bicarbonate (HCO3)	38 mmol/L (Normal: 22-29 mmol/L)
Blood Urea Nitrogen (BUN)	30 mq/dL (Normal: 7-26 mq/dL)
Creatinine (Cre)	0.6 mq/dL (Normal: 0.7-1.3 mq/dL)
Glucose (Glu)	151 mg/dL (Normal: 60-140 mg/dL)
Calcium (Ca)	11.2 mg/dL (Normal: 8.4-10.2 mg/dl)

**Figure 1:**
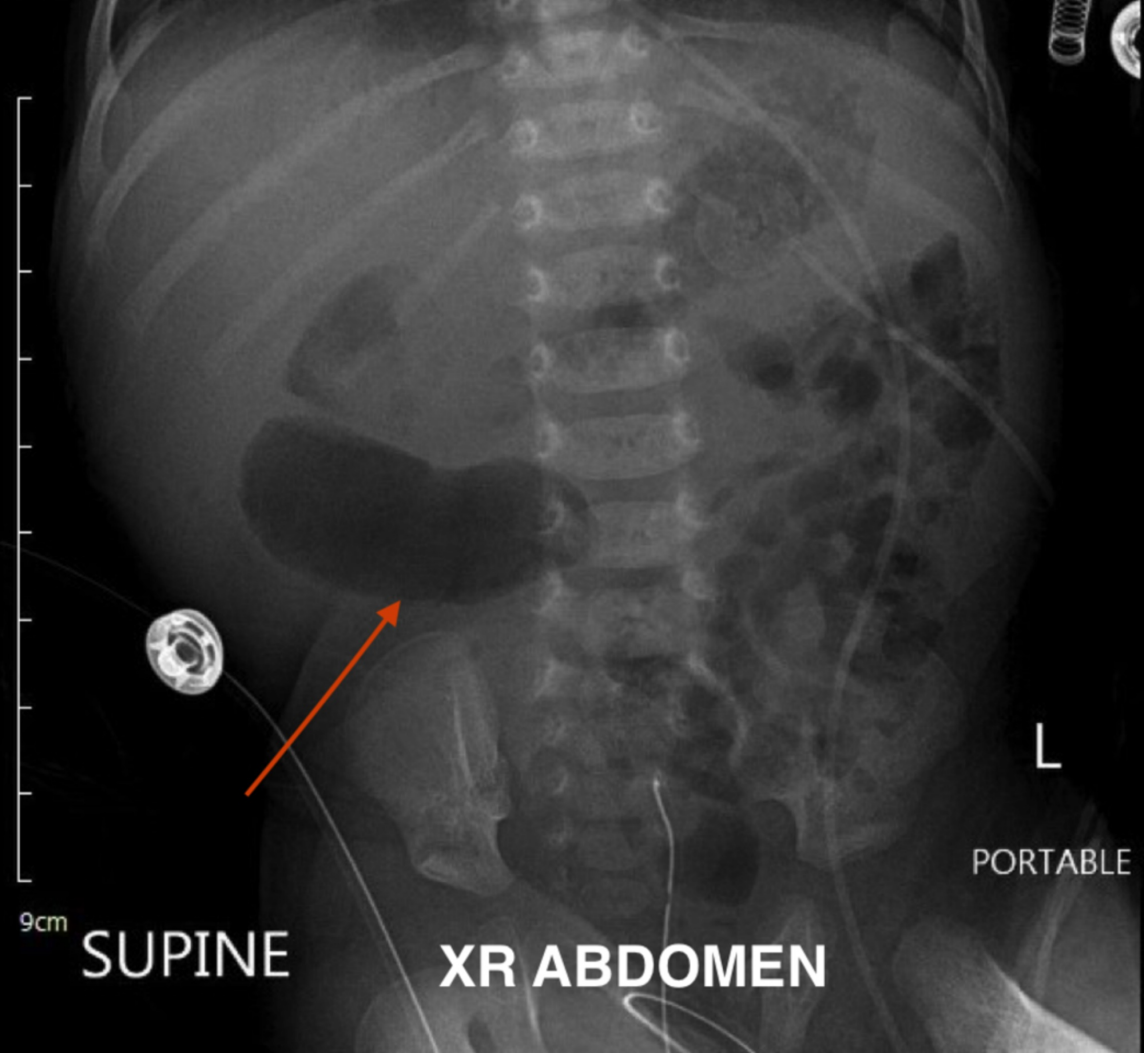
Abdominal X-ray with dialated loops of bowel

The patient was then given 0.9% normal saline (NS) bolus and maintained on intravenous fluids D5 ½ NS with 10 mEq/L of potassium chloride. The patient had two recurrent episodes of “yellow” vomitus and an episode of red-currant jelly vomit. Oral feeds were held and a nasogastric tube inserted. The patient's initial abdominal X-ray showed stool in the bowel without free air or any sign of obstruction. A second abdominal X-ray showed prominent loops of bowel in the right side of the abdomen (Figure 1).

**Figure 2:**
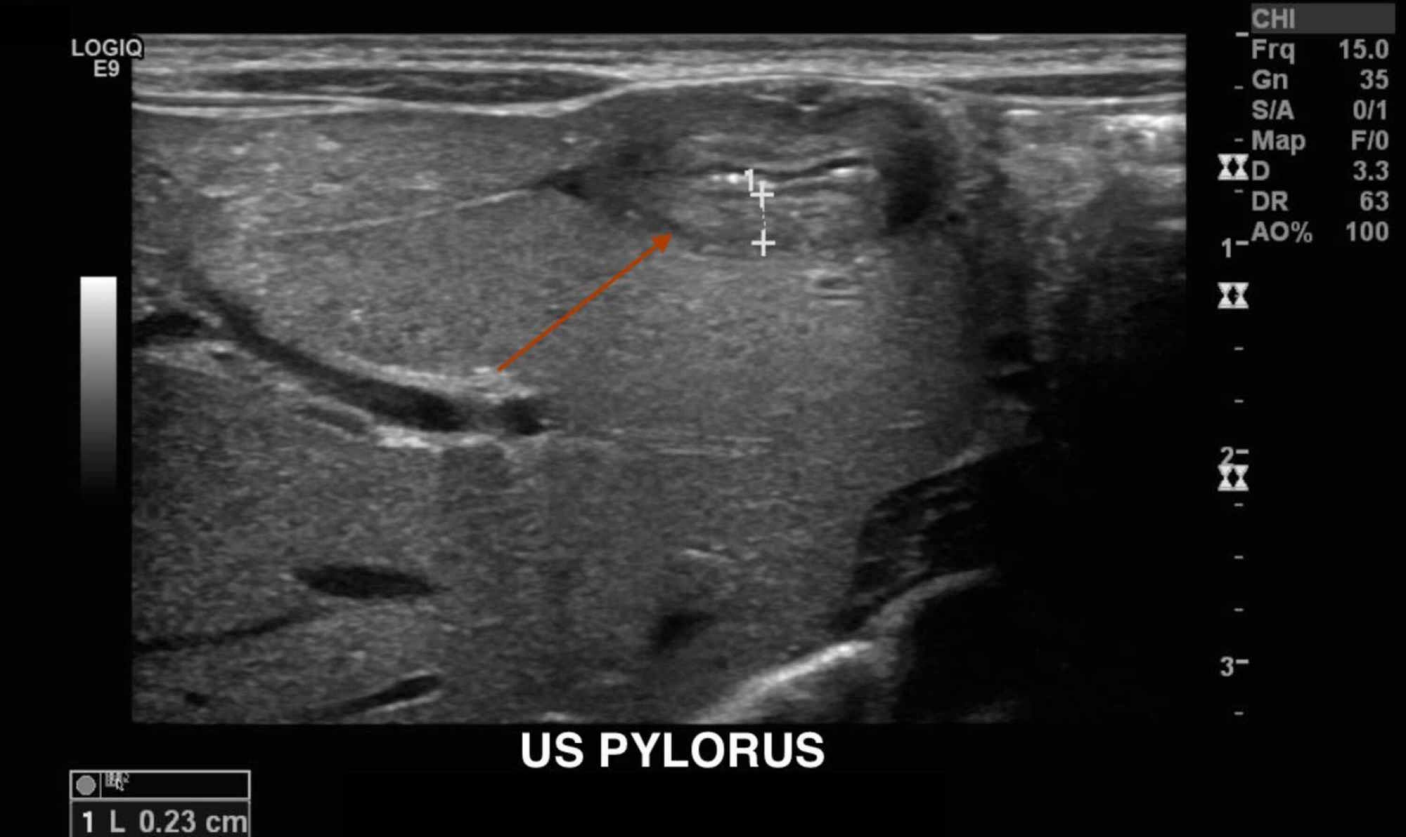
Ultrasound of the pylorus

On hospital day two, an ultrasound of the abdomen and pylorus was performed to rule out pyloric stenosis but the findings were inconclusive due to persistent vomiting during the study (Figure 2).

**Figure 3:**
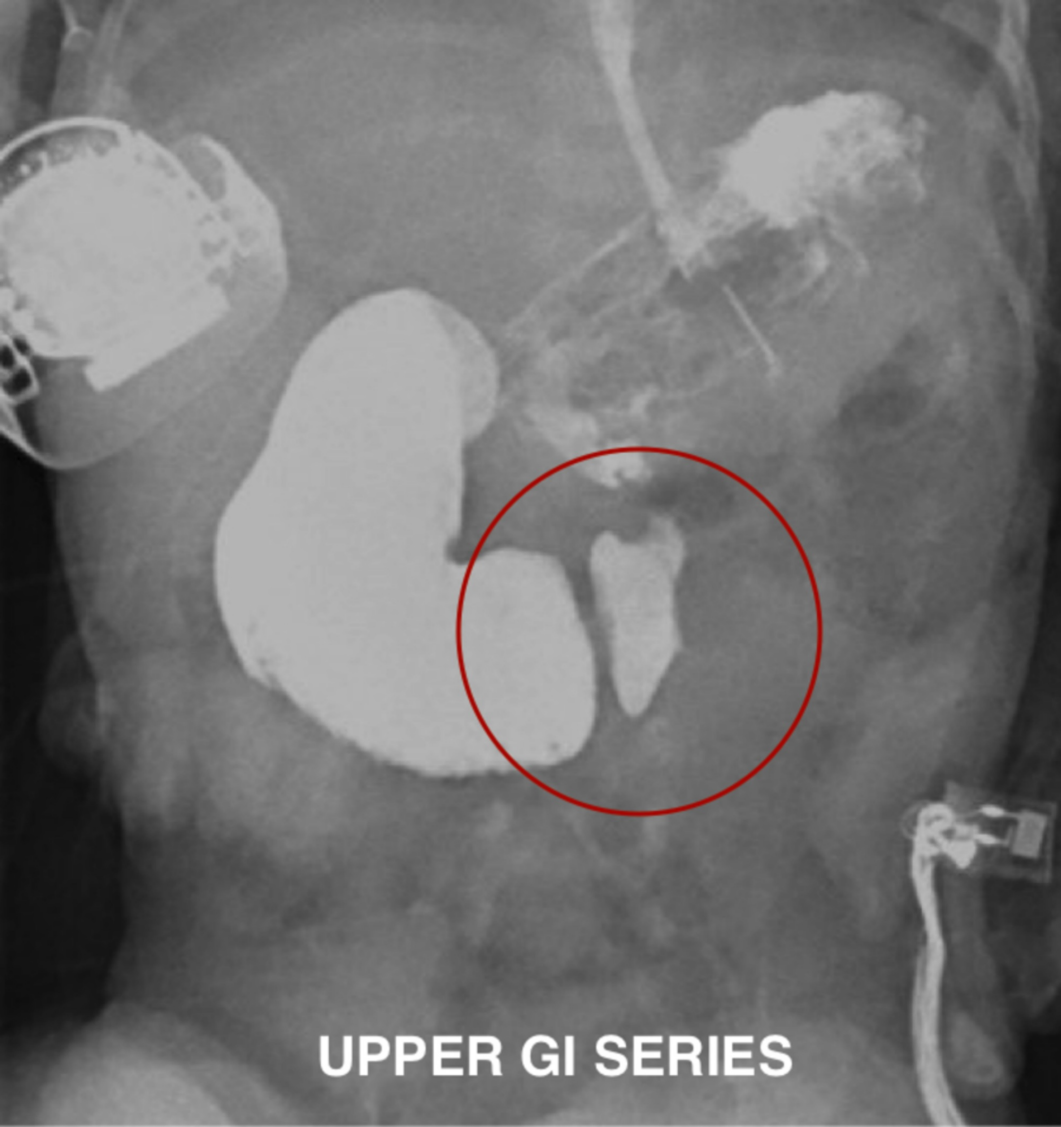
Upper gastrointestinal (GI) series showing stenosis at the level of the duodenum

An upper gastrointestinal (GI) series was then performed which revealed a partial obstruction at the level of the duodenum (Figure 3).

The patient underwent an exploratory laparotomy on hospital day five. Exploration revealed stenosis of the third part of duodenum with mild adhesions to pancreatic tissue. There was no evidence of pyloric stenosis. The adhesions were lysed and a duodeno-duodenostomy was performed, approximating the second and fourth portions of the duodenum. The patient received inpatient post-operative care and was subsequently discharged home to the mother following recovery.

## Discussion

The initial evaluation of an infant with suspected proximal bowel obstruction should focus on identifying the obstruction and on differentiating non-emergent congenital duodenal obstruction from emergent causes (e.g. malrotation with midgut volvulus) given their devastating consequences [2]. The vague and nonspecific clinical presentation of infants with congenital duodenal obstruction, particularly stenosis, can make identifying the condition particularly challenging. We aim to present a focused overview of this clinical entity, including a discussion on etiology, clinical presentation as well as diagnostic and treatment modalities. We will also highlight some of the diagnostic challenges we faced in this particular case as it pertains to the existing literature on duodenal obstruction and stenosis.

Etiology 

It is prudent to classify duodenal obstruction as intrinsic or extrinsic [3]. Intrinsic congenital duodenal obstruction refers to duodenal atresia, duodenal stenosis, and duodenal webs. The proposed etiology of intrinsic duodenal obstruction has been stated as failure of the duodenal lumen to recanalize during fetal development, which occurs at roughly 8-10 weeks in utero [4]. Duodenal atresia is the most common cause of congenital duodenal obstruction, with an incidence of roughly 1 in 10,000 live [5]. The second portion of the duodenum just beyond the ampulla of Vater is involved in greater than 70% of cases causing early-onset bilious emesis and the famous “ double-bubble” appearance on X-ray [6]. There is a high incidence of associated congenital anomalies, including trisomy 21 occurring in approximately 30% of patients [7] and biliary anomalies (including bifid common duct, choledochal cyst, and gallbladder agenesis) [8]. Duodenal webs cause 10%-30% of congenital duodenal obstructions [9]. 

An extrinsic duodenal obstruction such as malrotation can present with obstruction from either Ladd bands or midgut volvulus [10]. An annular pancreas, which can be as common as 1 in 1,000, can encircle the second portion of the duodenum causing an obstruction [11]. In the neonatal period, an annular pancreas is often associated with non-bilious emesis [7]. Another, albeit less frequent extrinsic causes of obstruction include preduodenal portal vein, superior mesenteric artery syndrome, duplication cysts, and replaced right hepatic artery [12]. In this clinical case, there was no evidence of an extrinsic compression. 

Clinical presentation

Duodenal stenosis denotes an incomplete obstruction and typically presents within the first 24-72 hours of life when feeding has commenced [9], however, the degree of stenosis ultimately dictates the age of presentation [13]. Patients, as expected, present later compared to duodenal atresia [7]. Due to the distal blockage, the most commonly associated symptoms are abdominal distension and vomiting [14]. Bilious vomiting is present in most cases of congenital duodenal obstruction, however, this can vary depending on the location of the stenosis. In our case, bilious emesis was anticipated as the obstruction was in the third portion of the duodenum, however, this was not a presenting feature in our patient again adding to the uniqueness of this clinical presentation. Depending on the age of presentation, symptoms can vary from recurrent vomiting, aspiration, or failure to thrive in infancy to gastroesophageal reflux and peptic ulceration later on [14]. Poor weight gain, signs of dehydration, minimal or no stooling are among other frequently associated symptoms of duodenal stenosis [14]. In our patient, small, spaced out feeds prior to admission allowed the presentation to be less characteristic and somewhat delayed. It should be noted that when full feeds were started on admission, the clinical symptomatology veered quite strikingly towards a mechanical obstruction with multiple episodes of emesis. Electrolyte imbalances are often observed with repetitive vomiting, which typically manifests as hypochloremic metabolic alkalosis. Such electrolyte imbalances in conjunction with non-bilious emesis are commonly associated with pyloric stenosis, which can present as early as the first week of life but typically manifests after three weeks until five months of life [15]. While our patient was marginally outside this range it was still a differential to be ruled out given the presenting symptoms. 

Diagnostic imaging

Prenatal ultrasound is the most reliable in the detection of congenital duodenal obstructions [16]. In the prenatal period, duodenal stenosis can manifest as polyhydramnios or dilated loops of bowel on fetal sonography [17]. Postnatally, the ultrasound is useful to confirm extrinsic etiologies of congenital duodenal obstruction such as annular pancreas, duplication cyst, and preduodenal portal vein [13]. It is also the diagnostic modality of choice for pyloric stenosis, which was ruled out in our patient [15]. Radiological investigation of suspected bowel obstruction begins with an abdominal radiograph that can change the management significantly if it does indeed demonstrate a true “double bubble” or multiple dilated bowel loops [7]. In our case, it was the latter as the typical “double bubble” sign was not apparent. Radiographs should be followed by an upper gastrointestinal series for further characterization [18]. In our patient, this was pivotal with regards to diagnosis and identifying the approximate level of obstruction. CT and MRI are not routinely used, but they are useful for visualizing vascular anomalies in older children being evaluated for nonspecific abdominal complaints [19].

Surgical repair

Immediate exploratory laparotomy is reserved for patients with suspected malrotation with or without volvulus [20]. Elective surgical correction is appropriate for duodenal atresia or stenosis. The current procedure of choice involves bypassing the obstruction with a duodeno-duodenostomy [9], as was performed in our patient. Postoperative care in terms of wound care, parenteral nutrition and appropriately resuming feeds are also of utmost importance.

This case was previously presented as an abstract at the Society of Critical Care Medicine. (Abstract: Carole M, Win K, Khin M, Kunal K, Louisdon P, Adebayo A. Duodenal Stenosis: A Diagnostic Challenge in an Otherwise Healthy Neonate With Poor Weight Gain. Society of Critical Care Medicine; February 17, 2019). https://journals.lww.com/ccmjournal/Citation/2019/01001/466__DUODENAL_STENOSIS__A_DIAGNOSTIC_CHALLENGE_IN.429.aspx

## Conclusions

Considering the vague and nonspecific clinical presentation of infants with congenital duodenal obstruction, even for the experienced clinician, this can prove to be a diagnostic challenge. Furthermore, as we have demonstrated by our case, it is often more challenging to diagnose incomplete duodenal obstruction in an infant. The clinical presentation varies with the degree of obstruction, age of presentation, while other factors such as feeding can modify the clinical presentation and make it more insidious. Considering the vagueness of signs and symptoms, a high index of suspicion coupled with sound clinical skills is warranted to diagnose this potentially catastrophic condition.
